# From seabed to sickbed: lessons gained from allorecognition in marine invertebrates

**DOI:** 10.3389/fimmu.2025.1563685

**Published:** 2025-04-10

**Authors:** Baruch Rinkevich

**Affiliations:** Department of Marine Biology, Israel Oceanographic & Limnological Research, National Institute of Oceanography, Haifa, Israel

**Keywords:** marine invertebrates, fusion, allorecognition landscape, chimerism, corals, tunicates, self/non-self recognition, organ transplantation

## Abstract

Despite decades of progress, long-term outcomes in human organ transplantation remain challenging. Functional decline in transplanted organs has stagnated over the past two decades, with most patients requiring lifelong immunosuppression, therapies that overlook the principles of self/non-self recognition and natural transplantation events in humans. To address these discrepancies, this perspective proposes that immunity evolved not as pathogen-driven but as a mechanism to preserve individuality by preventing invasion from parasitic conspecific cells. It further reveals that the concept of “self/non-self” recognition encompasses multiple theories with complex and often ambiguous terminology, lacking precise definitions. In comparisons, natural historecognition reactions in sessile marine invertebrates are regulated by a wide spectrum of precise and specific allorecognition systems, with transitive and non-transitive hierarchies. Using the coral Stylophora pistillata and the ascidian Botryllus schlosseri as models, it is evident these organisms distinguish ‘self’ from ‘non-self’ with remarkable accuracy across various allogeneic combinations, identifying each non-self entity while simultaneously recognizing selfhood through transitive allogeneic hierarchies. Their allorecognition offers an improved explanation for post-transplant outcomes by accounting for the natural dynamic, spatiotemporal evolution of selfhood. To bridge natural (in invertebrates and humans alike) and clinical transplantation phenomena, the ‘allorecognition landscape’ (AL) metaphor is proposed. This unified framework conceptualizes self/non-self recognition as shaped by two dynamic continuums of ‘self’ and ‘non-self’ nature. Throughout the patient lifespan, the AL represents diverse and transient arrays of specific ‘self’ and ‘non-self’ states (including reciprocal states) that shift over time in either recognition direction, requiring adaptable clinical strategies to address their evolving nature.

## 1 Introduction

Human organ transplantation represents a pinnacle of modern medicine, integrating advancements in immunology, genetics, pharmacology, and surgery into a highly successful discipline. Driven by the goal of extending life and improving human well-being, this field focuses on restoring functions through the deliberate replacement of damaged organs. Started about seven decades ago (1), organ transplantation has become a routine part of medical practice worldwide, often celebrated in mainstream media for its advancements. Yet, despite significant progress in unraveling the complex immune cascades and molecular interactions involved in transplantation, major challenges remain. Long-term outcomes for transplanted organs have seen little improvement, with functional decline rates remaining largely unchanged over the past two decades (2, 3), while most patients depend on lifelong immunosuppressive therapy, as withdrawal typically leads to allograft rejection. Clearly, improving long-term graft survival necessitates a deeper understanding of transplant injury mechanisms, alongside innovative research approaches and fresh perspectives that could drive transformative advancements in knowledge, practices, and technologies. Here I emphasize the importance of exploring allogeneic mechanisms underlying ‘self’ vs. ‘non-self’ recognition, extending beyond the conventional focus on mammalian systems.

Historically, organ rejection has been attributed primarily to adaptive immunity, including T-cell-mediated and antibody-mediated rejection. However, recent studies have uncovered the critical role of innate immunity, such as missing-self activation of natural killer (NK) cells and monocyte-driven allorecognition (4, 5). These findings underscore the importance of innate immunity in initiating early immune responses to transplanted allografts and contributing to late-stage chronic rejection. Additionally, they challenge the long-standing immunological paradigm that regards innate immunity as merely a downstream effector mechanism activated by adaptive immune responses during graft rejection (5, 6). While the adaptive immune system, primarily evolved for infection defense, is both necessary and sufficient for transplant rejection, the specific pathways of innate immunity involved remain poorly understood. Notably, rejection-associated alloimmunity appears largely independent of the signaling mechanisms underlying antimicrobial immunity (7). A similar ambiguity surrounds the mechanisms of graft-versus-host disease (GVHD), a major contributor to morbidity and mortality following allogeneic hematopoietic stem cell transplantation (8).

Yet, current treatment approaches primarily target adaptive immune responses, with limited attention to innate immunity (6). This highlights the need for a deeper understanding of innate immunity in organ transplantation and the development of innovative approaches to address acute and chronic organ rejection effectively. A refined scholarly approach could shift focus from detailed molecular pathways and cellular mechanisms of rejection to exploring the fundamental processes of ‘self/non-self’ recognition. Adopting this view, studying the natural transplantation in marine invertebrates and the semi-allogeneic nature of vertebrate pregnancies offer promising avenues. Such studies may uncover universal principles, shed light on the evolutionary roots of alloimmunity, and reveal homologous kinships across species, ultimately transforming our understanding and approach to.

## 2 The evolutionary roots for the immune system

Defense against microbial pathogens is a universal trait among all metazoans. In invertebrates, innate immunity serves as the primary defense mechanism, and many of its features have been conserved, in various forms, within vertebrates (9, 10). The hallmark of innate immunity is its reliance on germline-encoded receptors to identify harmful elements, whereas vertebrate adaptive immunity depends on gene rearrangement to generate its repertoire. Despite their differences, both types of immune systems participate in a wide range of biological processes (9–11), while employing diverse tools to combat pathogens. This has led to the dominant paradigm, reflected in immunology textbooks, that immune recognition and its associated effector mechanisms evolved primarily to combat infectious agents. The adaptive immune system’s effectiveness in neutralizing pathogens supports this view. However, evidence suggests that pathogens are not necessary to explain the high levels of polymorphism observed in immune systems (12). Additionally, all vertebrates and studied invertebrates exhibit allorecognition, using their immune systems to effectively reject allografts. Interestingly, this phenomenon does not naturally occur in adult vertebrates, presenting an intriguing evolutionary paradox (10).

To address this evolutionary paradox, we can explore alternative perspectives that challenge the prevailing view that vertebrate immunity evolved primarily to combat pathogens. One possibility is that vertebrate innate immunity may have originally served a different function in ancestral organisms. It may persist today as a relic or vestige of ancient systems that became redundant with the emergence of adaptive immunity (13, 14), or as an “evolutionary rudiment” whose sole role is to manage infections until the more robust adaptive immune response is activate (15). Another perspective suggests that vertebrate adaptive immunity may have co-opted an ancient polymorphic gene family encoding cell surface interaction molecules (16). For instance, molecules with multiple Ig-like domains, which emerged early in eukaryotic evolution, are present in yeast a-agglutinin cell wall proteins (17), in the extracellular domain of receptor tyrosine kinase in the marine sponge *Geodia cydonium* (18), or that marine invertebrates from disparate phyla reveal highly conserved immune machinery (19). A third perspective posits that the immune system’s original function was to preserve individuality. This involved preventing the intrusion of conspecific alien cells into the soma and germline or eliminating newly introduced somatic mutations. An organism incapable of controlling the proliferation of somatic variants or alien conspecific cells could effectively be parasitized by these lineages. In this framework, pathogen defense may have evolved later, giving rise to the diverse immune phenomena observed today (9, 10). This perspective, prioritizing individuality preservation, necessitates acknowledging naturally occurring transplantation events in vertebrates. It challenges the conventional view that vertebrate and human allograft reactions are purely artificial phenomena. Examples of natural transplantation in humans include fetal implantation, early fusions of dizygotic twins, and the persistence of fetal cells in the maternal bloodstream decades postpartum (9, 20, 21). I align with this third proposal.

Vertebrates robustly reject any allogeneic transplanted tissue, demonstrating strong defenses against events that do not occur naturally, yet fail to prevent the lifelong establishment of various natural transplantation events. Therefore, rather than the typical comparison of invertebrate and vertebrate immune systems based on innate versus adaptive responses to pathogens, greater focus should be placed on evaluating allorecognition as a potential shared foundational system underlying the evolution of diverse immune mechanisms. Organ transplantation, while not a natural phenomenon, should be considered within the broader context of innate allorecognition responses and their unresolved mysteries.

## 3 Self versus non-self recognition

A prominent perspective on the evolutionary pressures shaping the immune system is the concept of immunologic surveillance, introduced over six decades ago (22). This framework posits that host organisms are perpetually exposed to external pathogenic threats, driving the evolution of immune systems to distinguish and defend against harmful intruders. As a result, immunity is often framed as the ability to differentiate “self” from “non-self,” serving as a foundational guideline in immunology. Yet, the “self/non-self” paradigm, while widely referenced, lacks inherent clarity and functions more as a guiding framework for exploring identity (23), at all levels of the ‘units of selection’ (24). Despite the precision of self–nonself recognition system (25), this concept remains entangled in semantic ambiguities, analogies, and complex theorizing, with limited clarity provided by scientific discourse (26).

The diverse expressions of “self/non-self” recognition in mammalian systems and the extensive study of this topic have led to years of detailed examination, resulting in numerous viewpoints and the emergence of complex terminology. Without delving into an historical account, the two decades following Burnet’s (22) suggestion of self-recognition in marine invertebrates, saw a proliferation of perspectives on the “self/non-self” paradigm in vertebrates. These included Janeway’s (27) theory that the immune system evolved to distinguish “infectious nonself” from “noninfectious self”, the ‘peptidic self model (28), the “liquid self” (29), the ‘high determinant density’ idea for alloreactivity (30), the Kärre’s ‘missing self’ model (31) and Versteeg’s (32) proposition that the immune system incorporates elements for recognizing both self and nonself. Other perspectives include Daunter’s (33) distinction between “self-foreignness” and “foreignness per se” and Matzinger’s (34) ‘danger signals’ theory, which suggests that immune responses are triggered not by “non-self” or ‘‘infectious non-self’’ but by the detection of ‘‘danger signals’’ by the host. These and other diverse ideas highlight the complexity and ongoing evolution of our understanding of immune system function. Additionally, popular yet often ambiguous terms such as ‘pattern recognition receptors’ (PRRs), ‘pathogen-associated molecular patterns’ (PAMPs), and ‘damage-associated molecular patterns’ (DAMPs) have emerged in discussions of self/non-self recognition. While widely adopted, these terms frequently lack precise definitions, reflecting the inherent ambiguity and implicit assumptions in scientific terminology. Moreover, in recent years, the traditional discussions on immune self versus non-self mechanisms have expanded to include processes such as the discrimination involved in spacer selection for palindromic repeats (CRISPR) and CRISPR-associated (Cas) proteins in prokaryotes (35, 36), to anti-cancer therapies (37–41), vaccine development (42), autoimmune diseases (43), the recognition of foreign nucleic acids (44, 45), and towards artificial immune systems (46).

Historecognition systems are well-documented across various marine invertebrate phyla, especially among sessile organisms like sponges, cnidarians, bryozoans, and tunicates. For sessile marine invertebrates, physical space is often limited. As these organisms expand, they may come into contact with specimens of other species as well as non-kin conspecifics. These tissue-to-tissue interactions are often regulated by self/non-self recognition systems, where high levels of label diversity improve recognition accuracy. The distinction between ‘self’ and ‘non-self’ is made either by detecting the presence or absence of self-defining attributes or by identifying nonself-specific attributes (47, 48). To confirm the existence of alloimmunity in invertebrates, Hildemann et al. (49) proposed three key criteria: the expression of antagonistic reactions, demonstration of specific responses, and the ability to induce memory, all of which should be interrelated. Building on this, Janeway (50) introduced three additional criteria for a biological system to be classified as an immune system: the ability to precisely distinguish between self and non-self, the targeted generation of effector responses against non-self molecules, and the capacity to regulate these responses effectively. These criteria have spurred numerous studies across a wide range of invertebrate species and phyla. However, the concept of immune “self” in these studies, as well as in the broader literature, remains undefined due to its conceptual and mechanistic ambiguity [but see some attempts (51, 52)].

**Figure 1:**
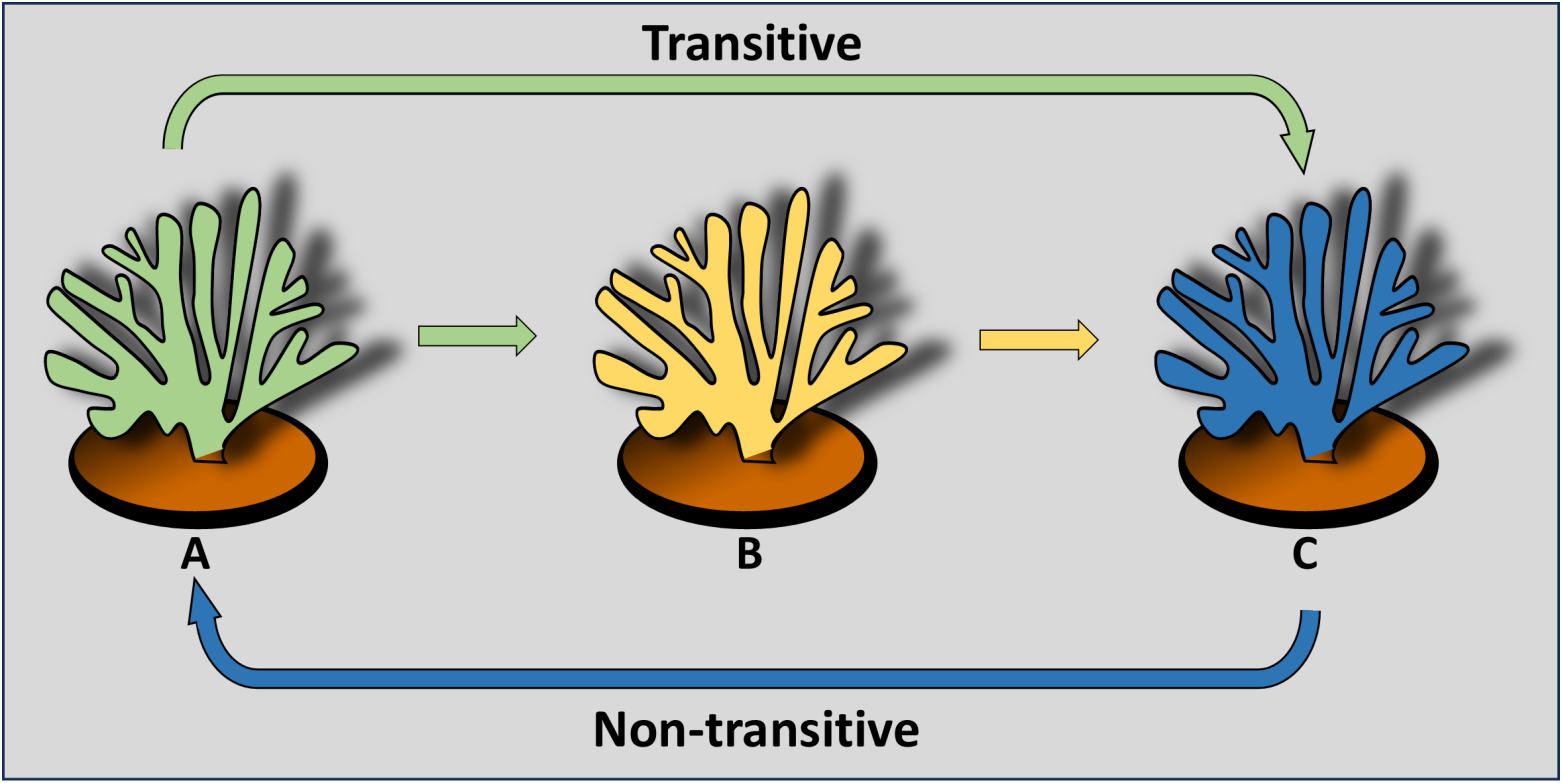
A cartoon depicting the simplest transitive (linear) and non-transitive (circular) allorecognition relationships among three conspecifics (the various colors) of the hermatypic coral *Stylophora pistillata* (**Figure 2a**). The colored arrows depict directionality and hierarchy of rejection outcomes.

Specific responses lead to allorecognition transitivity among conspecifics when more than two partners are involved. The simplest scenario reflects three conspecifics (A, B, C) that are tested for fusion/rejection phenomena. Transitivity is confirmed when (= for fusion; ≠for rejection): A = B and B = C, then A = C, or when A = B but A ≠ C, then B ≠ C. Nontransitive relationships occur when A = B, A = C, but B ≠ C. Specific hierarchies are established when A > B and B > C, leading to A > C for a linear hierarchy, or A < C for a circular hierarchy (**Figure 1**) (47, 53).

## 4 What can allorecognition in marine invertebrates teach us?

**Figure 2:**
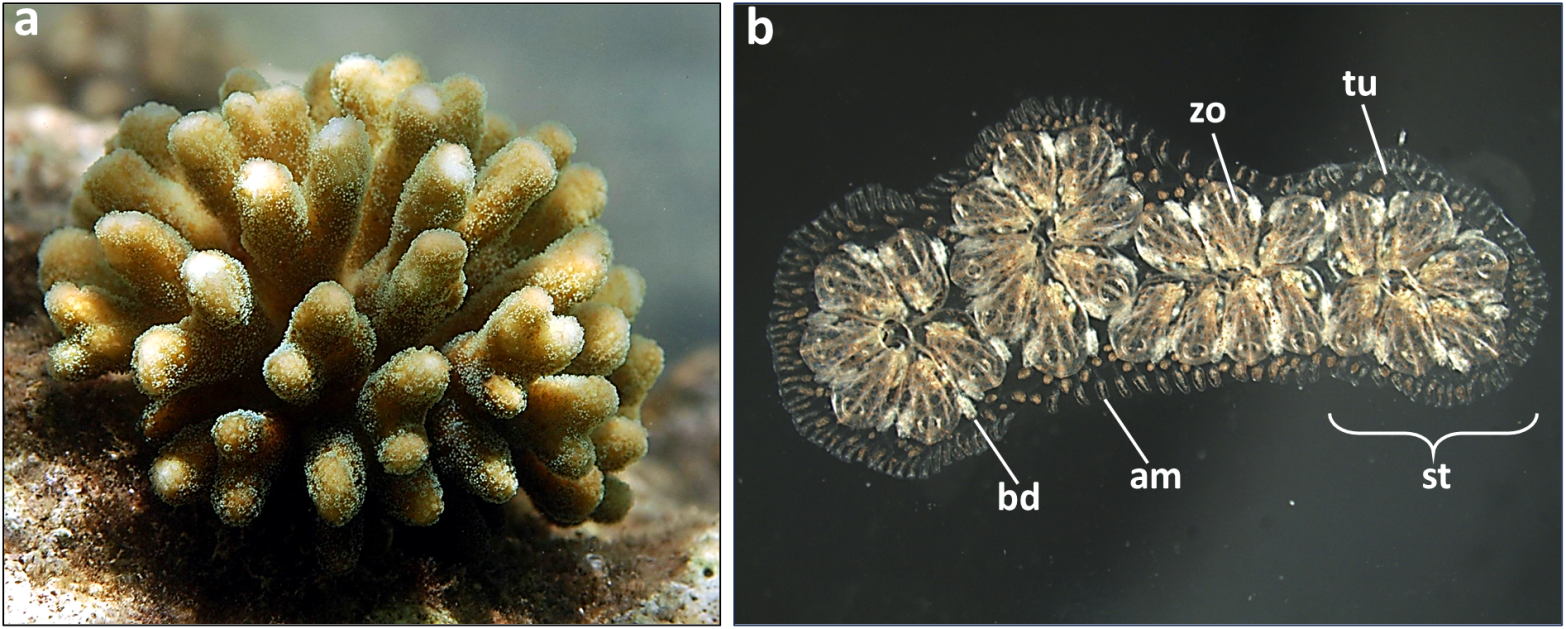
The two representative marine invertebrates: **(a)** a colony of the branching coral *Stylophora pistillata* growing in the field; **(b)** a colony of the tunicate *Botryllus schlosseri* growing in the laboratory on a glass slide. Zooids (zo, each 2 mm long) form star-shaped clusters (system, st), each with a centered shared atrial siphon. The zooids are embedded in a transparent tunic(tu) containing vessels and terminal ampullae (am) of the colonial circulatory system. Buds (bd) are partially covered by adult zooids.

To clarify the concept of allorecognition in marine invertebrates, I will elaborate alloimmunity in two representative species, one from the anthozoan basal phylum, the common Indo Pacific branching coral *Stylophora pistillata* (**Figure 2a**) (54) and the second from the highly evolved urochordates, the cosmopolitan colonial ascidian *Botryllus schlosseri* (**Figure 2b**) (55).

**Figure 3:**
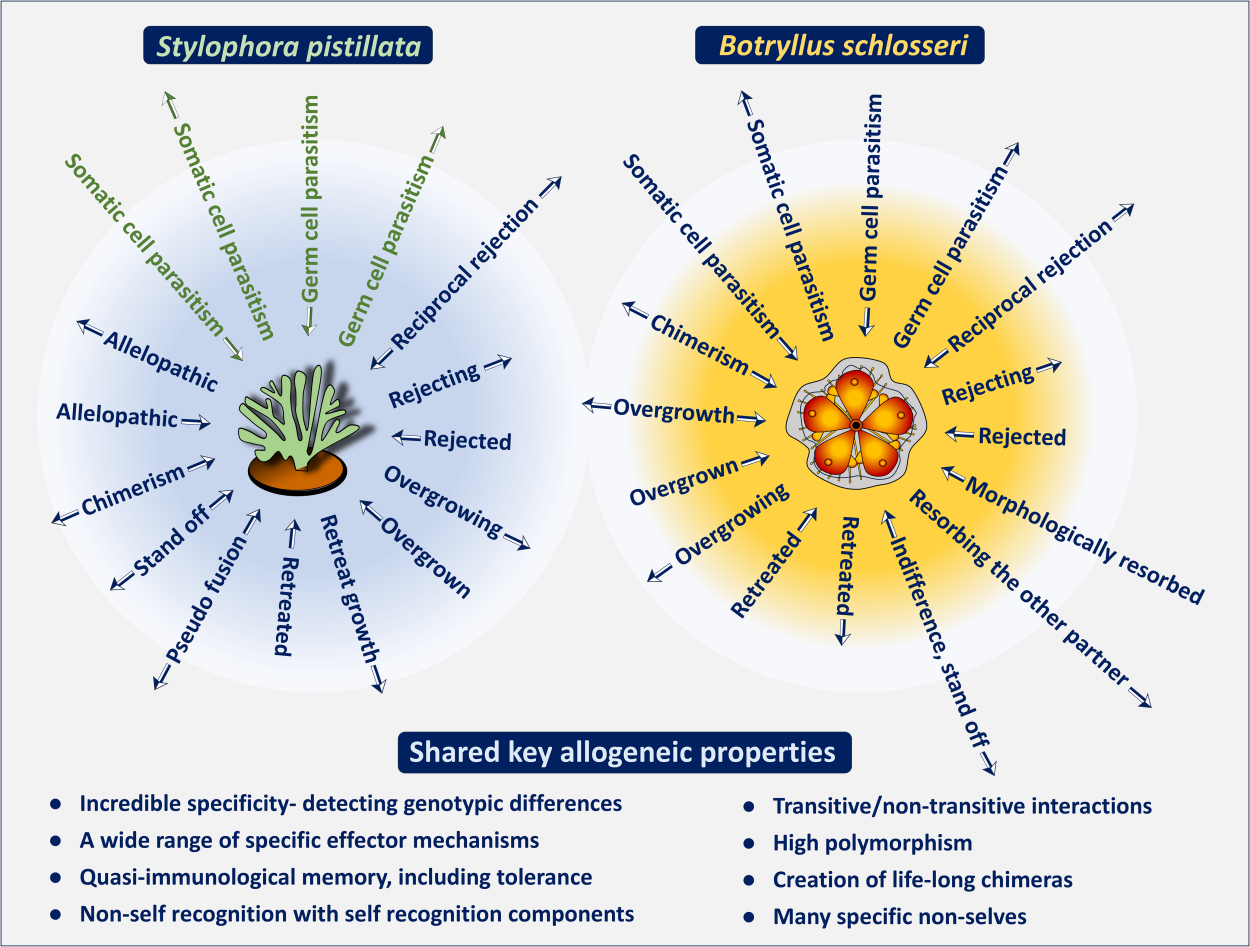
A schematic illustration showcasing the remarkable diversity and precise specificity of historecognition in the Cnidaria (represented by *Stylophora pistillata*; left panel) and in the Tunicata (represented by *Botryllus schlosseri*; right panel). Colonies of these marine invertebrates are naturally encountered in various allogeneic responses (arrowheads reveal hierarchies for the effector arms). A single invertebrate genotype is not restricted to a single mode of interaction during allogeneic encounters, thus its extensive repertoire of effector mechanisms allows for precise and specific responses to an unlimited range of 'nonself' attributes. At the bottom: shared key allogeneic properties. The *S. pistillata* green allogeneic interactions- suggested, not yet approved.

While the genetic background of *S. pistillata* has not yet been fully characterized, it is known that adult genotypes never fuse, and fusion occurs only during early life stages (0–4 months old spats). Juvenile colonies with shared parentage (kin) display higher fusion rates compared to unrelated colonies, emphasizing the role of genetic relatedness in fusion outcomes (56–58). Iso-grafts always fused where allografts resulted with a wide range of incompatible responses (**Figure 3**) (59–61). In *B. schlosseri*, both adults and young colonies can fuse. This allorecognition is genetically controlled by a single haplotype, called *BHF (* 62), which determines compatibility and allows vascular fusion among individuals. Incompatibility, on the other hand, triggers inflammatory rejection responses. The *BHF* locus exhibits extraordinary polymorphism, with 100–300 codominantly expressed alleles per population. A colony can fuse with another colony that shares at least one of its two *BHF* alleles, even if the second allele or the rest of the genome differs. However, colonies that reject each other lack any shared *BHF* allele, even if their genomes are highly similar (9, 63–66).

As proposed by Hildemann et al. (49) the demonstration of specific activity is a key criterion for establishing allorecognition, which is an inherent feature in both representative species. In *S. pistillata* [as well as in other coral species, e.g (53, 60, 67)], studies have shown the nontransitive nature of their effector mechanisms. In these nontransitive hierarchies, a colony that dominates in one interaction may be subordinate or equal in aggression to another colony that underperforms in the previous interaction (**Figure 1**). Additionally, colonies could specifically distinguish between neighbors and respond differently to allogeneic and xenogeneic challenges (**Figure 3**) (58–60, 68–70). For xenogeneic interactions, field observations revealed that degraded tissues at contact points between *S. pistillata* and adjacent coral species were marked by aggression hierarchies through highly specific aggressive outcomes, with *S. pistillata* often ranked as an inferior competitor (71). In allogeneic interactions, grafting assays conducted both *in situ* and *ex situ* confirmed that genetic background influences intraspecific interactions and revealed both transitive and non-transitive hierarchies (59–61). Allografts elicited a variety of effector mechanisms, with a single *S. pistillata* genotype reacting differently and specifically to various conspecific genotypes, indicating precise directionality in its effector mechanisms. This intricate pattern of incompatibility in *S. pistillata* reflects a ‘non-self recognition’ system, as genotypes can detect even subtle differences among closely related kin, exhibiting genotype-specific responses and a wide range of cellular and morphological reactions (**Figure 3**) (47). In contrast, isogeneic fusions reflect ‘self recognition’, separate from the ‘non-self recognition’ seen against conspecifics, indicating discrete recognition alternatives governed by the complex genetic makeup of the interacting partners. Furthermore, the directionalities of allogeneic effector arms in *S. pistillata* were highly consistent and reproducible (60, 61), representing internal, specific outcomes of recognition and not the result of external biological cues such as predation or competition. These organisms, which lack circulatory systems or specific immune cells, demonstrate remarkable precision in distinguishing their isogeneic, allogeneic, and xenogeneic environments.

**Figure 4:**
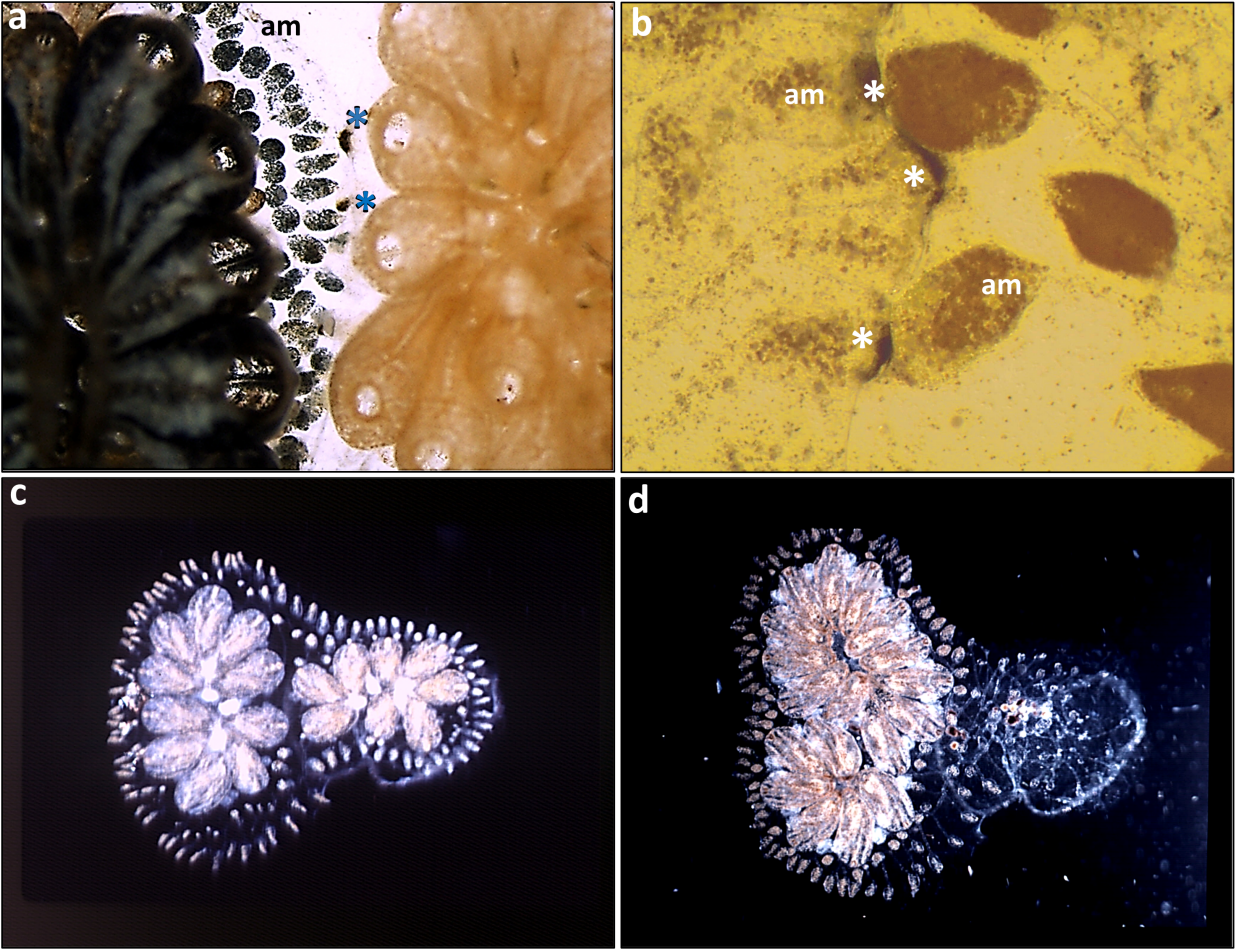
*Botryllus schlosseri* allorecognition. **(a, b)** Non-self recognition: **(a)** two PORs at contacting ampullae in the left colony tunic, marked by blue asterisks. **(b)** a close up of non-self recognition with 3 PORs at contacting ampullae in the left colony tunic, marked by white asterisks. **(c, d)** Resorption of the right partner in a chimera: **(c)** two weeks following chimera formation between two compatible young colonies. The left colony with 10 zooids, the right colony with 8 zooids. **(d)** several months thereafter. The right colony is completely resorbed, the left colony with two systems of functional zooids. am= ampullae.

As in *S. pistillata*, studies on *B. schlosseri* (**Figures 3**, **4A, B**) have shown that colonies can distinguish between neighbors and respond differently to allogeneic and even to xenogeneic challenges (including phenomena such as reciprocal or unilateral rejections, indifference, retreat growths, fusion, colony resorption, somatic/germ cell parasitism, and more), governed by nontransitive and transitive hierarchies of effector mechanisms with highly consistent and reproducible outcomes, as well as genotype-specific effector mechanisms targeting specific conspecifics (9, 63, 72–75). In allogeneic rejection cases, results (72) further revealed that a complete repertoire of points of rejection (PORs; **Figures 4A, B**) was established within 10 days, yet not all ampulla-ampulla interactions developed PORs. Additionally, cases of indifference, where ampulla-ampulla contacts did not lead to any rejection, were consistently observed in specific pair combinations, with their frequency increasing in repeated testing rounds, suggesting that the rejection phenomenon aligns with the characteristics of a low responder (72). These findings are compared with aspects of tolerance in mammalian systems. Following fusions between allogeneic conspecifics, partners in the chimeras are morphologically eliminated (the resorption phenomenon; **Figures 4C, D**). Fusions between compatible *BHF* genotypes reflect the ability for ‘self recognition’, while aggressive phenomena in the chimera elicit components of ‘non-self recognition’, as demonstrated by the rejection outcomes (**Figures 4A, B**) developed between non-compatible *BHF* genotypes.

Bypassing the usual interaction site (the extended ampullae) through the transplantation of zooids between *BHF* -incompatible pairs (76), revealed that: (1) instead of the typical tissue rejection (necrosis) observed during natural contacts at peripheral blood vessels, transplanted tissues were eliminated morphologically within a few days, consistent with the normal weekly developmental growth of the colony (76); and (2) donor-recipient chimerism was established after the complete removal of transplanted tissues. These results indicate that *BHF*-based allorecognition in *B. schlosseri* occurs exclusively at the ampullae, and once cells bypass this site, they can survive and proliferate in the host colony (76).

## 5 What can chimerism in marine invertebrates teach us?

Chimerism, the phenomenon that a single organism possesses cells of more than a single genotype of the same species, stands out as a crucial ecological and evolutionary mechanism, influencing the life history traits of protists, metazoans, and even humans (24, 25, 77–79). Clearly, natural chimerism is directly associated with allorecognition, the self/non-self recognition (21, 25, 48, 64, 77). In numerous instances, including in algae (80), invertebrates (58, 81, 82), and vertebrates, such as human (21, 83), chimerism occurs only briefly during early developmental stages. As in humans, fusion and chimera formation in *S. pistillata* can occur only during early life stages (0–4 months young colonies (57, 58),). In *B. schlosseri*, colonies may fuse upon contacts in any stage of their life span (47, 65, 73, 74). Chimerism thus reveals limitations or failures in the effectiveness of self/non-self recognition mechanisms. While humans reliably reject allogeneic transplanted tissues in iatrogenic settings, they cannot prevent the lifelong establishment of natural transplantations that result in chimerism (21, 83–85).

Tissue transplantations and chimerism in *S. pistillata* and *B. schlosseri*, while likely underrecognized in nature, have raised important questions about the diverse costs and benefits associated with the chimeric state. The literature highlights chimerism as a highly complex phenomenon with intricate biological and ecological implications, often described as a “double-edged sword” (77, 79), capable of circumventing both innate and adaptive immune responses. For *S. pistillata* chimeras, as with other coral species, chimerism represents a partnership between allogeneic individuals, conferring various advantages (56, 79, 86–89), which may explain why natural fusions among conspecific corals are common. Fusion between colonies offers chimeric organisms an immediate survival advantage by facilitating rapid size growth. Chimerism is believed to be a crucial strategy for enhancing survival during the vulnerable early life stages of corals and promoting growth, especially in these stages (56, 88, 90). Moreover, chimerism affects various biological and ecological traits, including increased reproductive success, earlier reproduction onset, improved competitive abilities during juvenile stages, reduced mortality rates for the entire entity (91), and greater resilience to adverse environmental conditions. This adaptability may act as an evolutionary rescue mechanism to mitigate the impacts of global climate change (87, 89). In turn, chimerism in *S. pistillata* bears impacts on pattern formation and polyp’s landscape (92).

In *B. schlosseri* chimeras, one of the partners or more partners (in chimeras made of multi-partners, multichimeras) are morphologically resorbed (73, 79, 93, 94), a process governed by multilevel hierarchical organization of allorecognition elements (95) and stress induced reversals (74). The rate of colony resorption in chimeras depends on the relative sizes of the colonies, with larger colonies requiring up to eight months and smaller ones as little as a week (96). Chimerism in this species can result in somatic and/or germ cell parasitism. Germ cell parasitism often leads to the complete reproductive dominance of one colony’s genotype, is asexually heritable, and frequently differs in directionality from somatic cell parasitism (65, 79, 97–99). While germ line parasitism is inherited through a pedigree, the somatic components of chimeric zooids can shift between genotypes in response to environmental changes (65, 100). This dynamic reorganization optimizes the chimeric entity by synergistically presenting the best-suited combination of genetic components under varying conditions (77, 87, 89, 100). Additionally, the deliberate co-settlement of histocompatible conspecific kin larvae (observed in *S. pistillata* and *B. schlosseri* (88, 96, 101);) significantly increases the likelihood of fusion compared to random settlement. This behavior raises important ecological and evolutionary questions regarding the costs and benefits associated with this widespread phenomenon.

Chimerism serves as a crucial ecological and evolutionary mechanism influencing the life history traits of metazoans, presenting in numerous forms and biological statuses (9, 20, 21, 25, 57, 65, 66, 73, 77, 79, 83, 87, 91, 94, 97, 99, 100). This intricate phenomenon functions as a “double-edged sword,” as while something provides benefits or advantages, it also has the potential for harmful effects or drawbacks. A recent analysis of chimerism (102) identified six dynamic and inter-changeable somatic forms (purged, sectorial, mosaic, mixed, micro, and multi-chimerism) and three active germline forms (mixed, male/female, and parasitic germline chimerism), based on the proportional contributions and spatial arrangements of chimeric partners within an organism. These variations in chimerism fall along two continua, ‘somatic cell chimerism’ and ‘germline chimerism’. Transitions between these states are fluid, with specific chimeric states capable of shifting into others over time. Thus, the chimeric state of an organism is part of a dynamic spectrum, where different states emerge and are replaced by others as the organism develops and adapts to its environment.

## 6 Natural transplantations in vertebrates

Allograft rejection is a strong response orchestrated by both the adaptive and innate immune systems (7), particularly through pathways that detect non-self and modified-self entities. While vertebrates consistently reject transplanted tissues from other members of the same species, they paradoxically tolerate various natural cell engraftments throughout their lives. These instances include phenomena such as cytomictical transplantation, fetal-maternal cell exchange, natural germ cell transplantations, transmissible allogeneic tumors, and male-to-female cell transplantation, all of which illustrate the complex interplay between immune tolerance and rejection mechanisms [details in (20, 21, 77, 83, 85, 103)]. Notable, many cases of these natural transplantation events, including those related to pregnancy, are closely linked to disease outcomes (21, 77, 83). Nevertheless, throughout mammalian pregnancy, the mother’s immune system not only tolerates the immunologically foreign fetus but actively supports it, facilitating both embryo implantation and development. This phenomenon challenges the traditional self–nonself theory of immune recognition. Remarkably, the concentration of fetal cells in maternal blood steadily increases during pregnancy, reaching over 100 fetal cells per milliliter at parturition (104), highlighting a close relationship between fetal cell dynamics and embryonic development. Furthermore, fetal cells have been shown to persist and fluctuate in the maternal body for decades after childbirth, suggesting a long-lasting biological connection between mother and offspring (21, 83, 85, 105, 106).

From an evolutionary perspective, certain natural engraftments, such as fetal-maternal transplantation in mammals, are thought to be by-products of the functions developed in primitive immune components. These components contribute to developing embryos that are immunologically “educated”, by equipping them with effector mechanisms designed to eliminate pervasive somatic and germline variations. This perspective challenges the earlier notion that such processes were merely evolutionary vestiges (21). Understanding this immunological discrepancy, where alien transplants are supported rather than rejected, is crucial for uncovering the fundamental principles underlying natural transplantation phenomena and their diverse manifestations (107).

## 7 So, why transplanted organs are rejected?

Iatrogenic transplantation is the standard treatment for end-stage organ diseases, including those affecting the kidney, liver, heart, and lung. Advances in immunosuppressive therapies and medical care have significantly improved 1-year graft survival rates to over 90% for most transplanted organs. However, long-term graft survival remains a challenge, with transplant half-lives ranging from 8–11 years for kidneys to less than 5 years for lungs (1–3, 108).

The rejection of transplanted organs is fundamentally linked to the concept of self versus non-self recognition, a principle that has evolved over time (4, 5, 7, 109). Modern immunology offers various interpretations of the self–nonself theory (15, 22, 23, 26–28, 31, 34, 110), all based on the premise that the immune system originally evolved to protect the body against infections. Traditionally viewed as a defense mechanism against microbial threats, this raises the question of how the immune system recognizes parasitic entities while distinguishing them from the body’s own tissues, the core concept of ‘self’ versus ‘non-self’ recognition (26–28, 30, 31, 110). Additionally, it underscores the immune system’s remarkable ability to differentiate between various forms of “non-self” and adjust its responses accordingly (27, 110).

Natural transplantation in humans and other mammals occurs independently of iatrogenic transplantation and is inherently associated with the development of chimerism (20, 21). Chimerism is also evident in iatrogenic transplantation, where it is intentionally induced through the introduction of immune cells during organ transplantation. This artificial process parallels the natural implantation and development of a genetically ‘haploidentical’ fetus within the mother’s uterus. Yet the process is further more complex. Along pregnancy as an example, fetal microchimeric cells from one pregnancy are replaced by those from subsequent pregnancies, emphasizing the dynamic nature of chimeric status and the importance of microchimeric cell turnover for successful pregnancies (reviewed in (103)). In transplantation, an early major wound is made, where ischemia-reperfusion injury influences both the activation and response phases of alloimmunity. While these early events may obscure non-self recognition, akin to microbial infections, they fail to account for the persistence of alloimmunity long after the injury has resolved (110). It is also true that the process of iatrogenic transplantation is rarely analyzed within the context of natural transplantation in vertebrates or compared to analogous phenomena observed in marine invertebrates (20, 48, 78).

Allorecognition phenomena in marine invertebrates are marked by exceptional precision and specificity, as well as transitivity and a high degree of polymorphism (9–11, 25, 48, 49, 53, 57, 59–61, 66, 68, 73). Allorecognition assays performed on the branching coral *Stylophora pistillata* and the tunicate *Botryllus schlosseri* (as described above), reveal that they recognize ‘self’ and ‘non-self’ with remarkable accuracy when exposed to different allogeneic combinations. Unlike the concept of “self-recognition”, which categorizes all “non-self” entities as a single uniform alien (47), “non-self” recognition in these invertebrates allows for the individual identification of distinct ‘non-self’ allogeneic organisms (57, 59–61, 63, 67–69, 71–74). Further, in *Botryllus schlosseri*, ‘self’ recognition is so precise that fusion between allogeneic partners and chimera formation can occur with just one shared allele at the fusibility locus (9, 47, 48, 73), even when the second allele is identified as ‘nonself’. Thus, as noted by Neigel and Avise (53), a single marine invertebrate is not confined to a single mode of interaction during allogeneic encounters but instead responds adaptively based on the “properties of the system.”

Self-recognition among allogeneic marine invertebrates results in chimera formation, accompanied by both costs and benefits, as previously discussed (see also (9, 66, 73, 77, 83, 87, 97, 100)). If the immune system’s primary function was to maintain individuality by preventing the invasion of conspecific foreign cells into the somatic and germline tissues, or by removing newly formed somatic mutations, then human natural chimerism warrants further examination. In this context, it seems that the immune system’s original function has been compromised, leading to the complex and potentially conflicting (“double-edged sword”) effects of chimerism (20, 21, 77).

Iatrogenic transplantation bypasses the natural pathways that facilitate immune tolerance, pathways which are not yet fully understood, despite their associated costs, such as autoimmune diseases (84, 103, 105, 107). These natural processes, which enable successful transplantations in humans, involve complex mechanisms including substantial T helper and T regulatory cell activation, B cell involvement, and the innate immune system’s recognition of non-self or ‘damaged’ self through pattern recognition receptors. These receptors typically detect conserved microbial PAMPs, as well as theories like the missing-self theory and the danger hypothesis (reviewed in (5–7)). While the various self-nonself theories offer a useful framework for pre-transplant preparation, they fall short in explaining the diversity of post-transplant phenomena, primarily when compared with human natural transplantation events. In contrast, allorecognition patterns in marine invertebrates, such as *Stylophora pistillata* and *Botryllus schlosseri*, offer a more comprehensive explanation for post-transplant outcomes by accounting for the dynamic, spatiotemporal evolution of the immune self in response to environmental factors.

**Figure 5:**
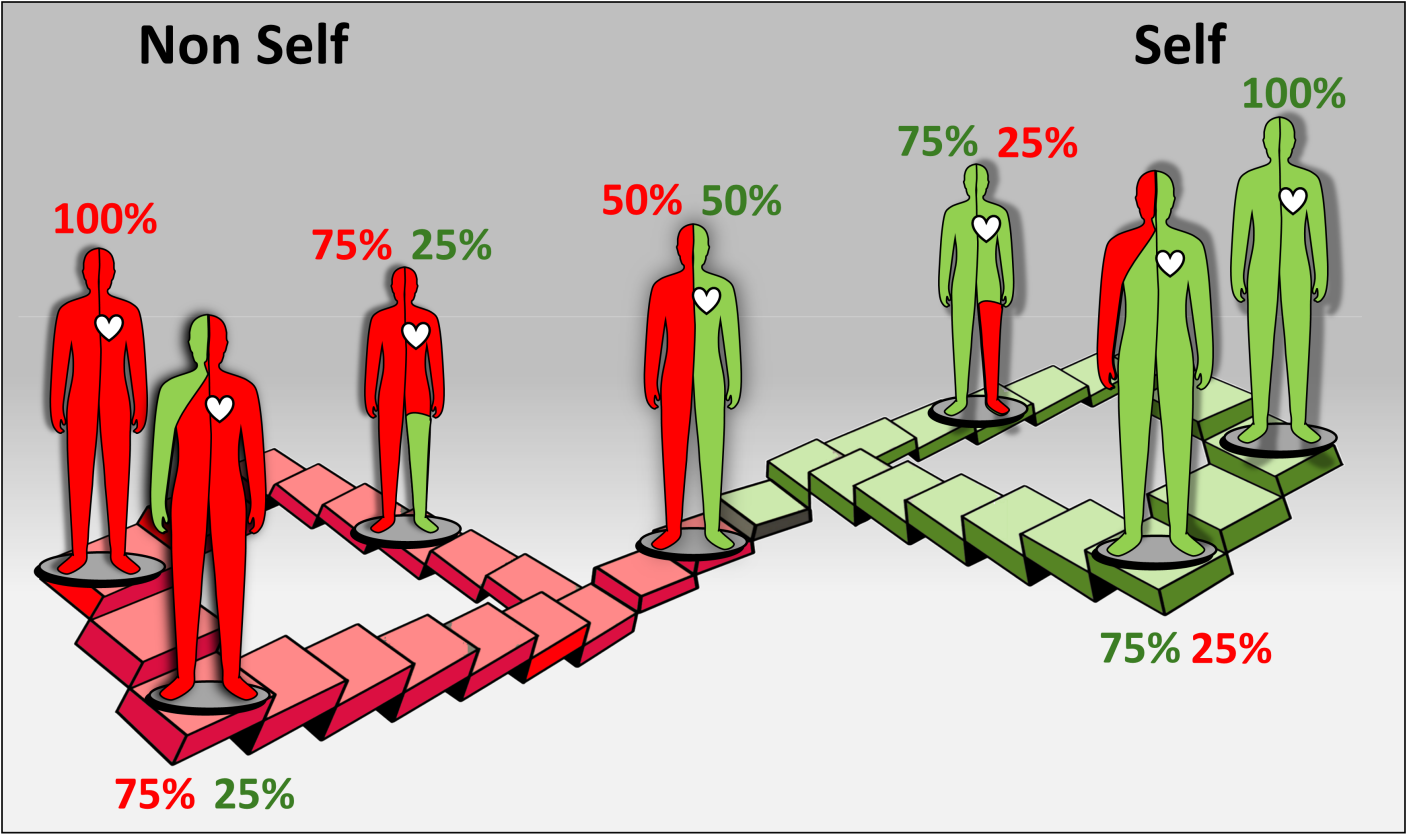
A schematic illustration of the evolving ‘allorecognition landscape’ metaphor and the shifting ‘self/nonself’. The figure illustrates the dynamic nature of immunological "self/nonself" recognition (distinct from effector mechanisms) in humans with transplanted organs. This process is represented as a unified allorecognition landscape, shaped by two recognition planes or continuums (depicted in red and green). Throughout an individual's lifespan, these continuums reflect diverse arrays of specific allorecognition states, including reciprocal states of 75:25%, 50:50%, and 25:75%. These recognition states are transient and can shift over time in either direction, transitioning into various states and requiring tailored clinical considerations.

Our critical evaluation of the mammalian and the marine invertebrates allorecognition processes, provide a unified conceptualization idea that the immune self is continuously changing, alternating between self and non-self statuses, highlighting the philosophical essence of its ongoing transformation (the proposed ‘allorecognition landscape’ metaphor; **Figure 5**), as further demonstrated above by discussions on allorecognition in marine invertebrates.

The clinical outcomes of transplant patients are highly variable, even with extensive knowledge of HLA molecules and immune mechanisms, as outcomes range from excellent to poor. Immune performance remains unpredictable, as some patients avoid rejection despite high-risk pre-transplant profiles, while others experience severe, unexpected rejection. Complications also vary widely among patients, both in type and sequence, highlighting our gap knowledge. This complex variability reflects the remarkable complexity, precision, and specificity observed in marine invertebrate allorecognition phenomena, including their intricate transitivity, high polymorphism, and ability to recognize in parallel multiple selves, each reacted distinctly by unalike effector mechanisms, and in different allogeneic combinations (9–11, 25, 48, 49, 53, 57, 59–61, 66, 68, 73). Even pregnancy that is believed to be a tolerant state because the fetus is not being rejected, is not always like that. We usually consider successful pregnancies when making this assessment, yet documentations exist for many unsuccessful fertilizations, implantations, and pregnancies represent in various ways the effects of various intolerant states (111).

The immunological ‘self/nonself’ is a key principle in immunology that serves as a fundamental framework for understanding how the immune system distinguishes and manages foreign entities, cells of related species and the body’s own components. This is illustrated by the metaphor of the ‘allorecognition landscape’, as illustrated in **Figure 5**. The interactions between a transplanted organ and the recipient’s body operate within two distinct, yet interconnected continuums of ‘self’ and ‘nonself’ recognition statuses, resembling an infinite ‘Escherian stairwell’ of selfhood. Each continuum features a complex array of precise and specific allorecognition elements, allowing the recognition state of the organ to fluctuate in response to environmental cues and the interaction of adaptive and innate immunity. These allogeneic states are dynamic and transient, capable of changing over time, which requires adaptable clinical strategies and considerations. The transition between these states can range from tolerance to complete rejection, potentially persisting throughout the patient’s lifespan. Thus, the ‘self’ and ‘nonself’ metaphors are not defined by fixed molecular recognition, rather, they embody a dynamic and ever-evolving allorecognition landscape that encompasses a wide range of states, from complete (100% in **Figure 5**) ‘self’ or ‘nonself’ recognition to myriad intermediate combinations where both recognition types coexist and function simultaneously to varying extents at any given moment.

It is important to recognize that the commonly employed anti-rejection therapies target immune effector mechanisms and clinical outcomes, rather than addressing the immune self/nonself metaphors. This current clinical approach reflects the broad suppression of the immune response without accounting for the redefinition of immune selfhood introduced by the transplanted organ. Thus, by providing a robust explanation of real-world chimeric phenomena with shared underlying structures, examining immunological scenarios through ecological and evolutionary perspectives, and exploring the extensive prevalence of natural transplantation (most notably in marine invertebrates), innovative clinical strategies for managing transplanted organ rejection may emerged.

## Data Availability

The original contributions presented in the study are included in the article/supplementary material. Further inquiries can be directed to the corresponding author.
